# Cardio-Ankle Vascular Index and Indices of Diabetic Polyneuropathy in Patients with Type 2 Diabetes

**DOI:** 10.1155/2017/2810914

**Published:** 2017-05-09

**Authors:** Akihiko Ando, Michiaki Miyamoto, Kazuhiko Kotani, Kenta Okada, Shoichiro Nagasaka, Shun Ishibashi

**Affiliations:** ^1^Division of Endocrinology and Metabolism, Department of Internal Medicine, Jichi Medical University, Tochigi 329-0498, Japan; ^2^Department of Laboratory Medicine, Jichi Medical University, Tochigi 329-0498, Japan; ^3^Division of Community and Family Medicine, Centre for Community Medicine, Jichi Medical University, Tochigi 329-0498, Japan

## Abstract

The cardio-ankle vascular index (CAVI) is used to test vascular function and is an arterial stiffness marker and potential predictor of cardiovascular events. This study aimed to analyze the relation between objective indices of diabetic polyneuropathy (DPN) and the CAVI. One hundred sixty-six patients with type 2 diabetes mellitus were included in this study. We used nerve conduction studies (NCSs) and the coefficient of variation of the R-R interval to evaluate DPN. We estimated arteriosclerosis by the CAVI. Simple and multiple linear regression analyses were performed between neuropathy indices and the CAVI. In univariate analysis, the CAVI showed significant associations with sural sensory nerve conduction velocity and median F-wave conduction velocity. Multiple linear regression analysis for the CAVI showed that sural nerve conduction velocity and median F-wave conduction velocity were significant explanatory variables second only to age. In multiple linear regression analysis for sural nerve conduction velocity among neuropathy indices, the CAVI remained the most significant explanatory variable. In multiple linear regression analysis for median nerve F-wave conduction velocity among neuropathy indices, the CAVI remained the second most significant explanatory variable following HbA1c. These results suggest a close relationship between macroangiopathy and DPN.

## 1. Introduction

A pandemic increase in type 2 diabetes leads to high morbidity and mortality because of its complications. A relation has been reported between macrovascular and microvascular impairment in type 2 diabetes [1–5]. There have been many studies concerning diabetic polyneuropathy (DPN) and its association with diabetic macroangiopathy [6–8]. Additionally, several studies have shown pathophysiological associations between DPN and macroangiopathy, as well as a similar onset time of DPN and macroangiopathy in the early phase of progression in diabetic complications [9–11]. Macrovascular impairment progresses through several phases, such as endothelial dysfunction, low vessel wall elasticity, and resulting structural sclerosis. Macrovascular damage together with microvascular change causes ischemia and hypoxia in neural tissues because of disordered endothelial function, arterial stiffness, and stenosis [12, 13]. This leads to dysregulated cardiovascular autonomic function. Loss of vessel wall elasticity and insufficiency of the peripheral circulation impair normal functioning of neurons. A prospective study showed that the incidence of DPN is associated with potentially modifiable cardiovascular risk factors, including elevated triglyceride levels and body mass index, smoking, and hypertension [14]. DPN as assessed by a 10 g monofilament was reported to be associated with an increased risk for cardiovascular events among individuals with diabetes [15]. Most previous studies on the relation between DPN and diabetic macroangiopathy were based on serum lipid markers, blood pressure, and neurological symptoms. Physiological assessment of arteriosclerosis, using pulse wave velocity (PWV) and intima-media thickness measurement of the carotid artery, is related to indices of diabetic autonomic neuropathy. These indices include the coefficient of variation of the R-R interval (CV_R-R_) [16–18] as well as indices of somatic neuropathy [19, 20].

Macrovascular impairment can be evaluated by arterial stiffness. PWV as a marker for arterial stiffness has become popular for its simple usability. However, application of PWV is limited by its dependence on blood pressure of the measurement point [21]. The cardio-ankle vascular index (CAVI) has been developed in Japan to overcome this limitation [22]. The CAVI can be determined from the stiffness parameter *β*, which represents the change in blood pressure required to expand the diameter of the artery. As a result of applying Bramwell-Hill's equation, the CAVI does not depend on blood pressure at measurement [23]. Recently, there have been two reports on the CAVI and DPN [24, 25]. One report showed that patients with DPN have a higher CAVI than those without DPN. Additionally, in multivariate analysis, this report showed that the CAVI was a significant determinant of DPN [24]. In the other report, the authors performed a retrospective, cross-sectional study of Korean patients with type 2 diabetes and showed that an increased CAVI was associated with DPN [25].

One of these previous studies defined DPN based on neuropathic symptoms, insensitivity of a 10 g monofilament, abnormal pinprick sensation, and the current perception threshold [24]. The nerve conduction study (NCS), which is the gold standard for objective diagnosis of diabetic neuropathy, was not used in this previous report [26]. In the other study, DPN was defined as a positive result of a neuropathy test (NCS, current perception threshold, and autonomic function test) [25]. The difference in CAVI values between those with DPN and those without DPN was examined. The current perception threshold used for DPN diagnosis was not totally objective and was dependent on patients' responses. A simultaneous change in the CAVI with NCS markers only was not demonstrated in this study [25].

In clinical practice, diagnosis and assessment of diabetic neuropathy depend on neuropathic symptoms and neurological examinations, which are subject to the patients' response, and vary according to the examiners. The most reproducible, reliable, and objective measures of DPN are NCSs [26]. However, NCSs can only assess large fibre neuropathy and they require some standardization [27].

In the present study, we aimed to determine the relationship between DPN and macroangiopathy by correlating neuropathy indices, especially NCS parameters, with the CAVI in patients with type 2 diabetes.

## 2. Materials and Methods

### 2.1. Study Design and Patient Population

We performed a cross-sectional study at Jichi Medical University in Japan. We recruited 207 inpatients and outpatients with type 2 diabetes from November 2011 to May 2015. Written consents were obtained from all of the patients. Finally, 166 patients were included in this study. Major exclusion criteria were as follows: age ≥ 75 years, renal failure requiring regular hemodialysis, those who had chemotherapy, alcohol or drug addicts, peripheral artery disease with an ankle-brachial index less than 0.9, neurodegenerative diseases (including Parkinsonism and dementia), cerebrovascular disease, and entrapment neuropathy. Patients who took *α*-blockers as an antihypertensive medicine were also excluded, because it was reported that *α*-blockers decreased CAVI value [28]. This study adhered to the tenets of the Declaration of Helsinki and was approved by the Institutional Review Board of Jichi Medical University.

The presence of neuropathy was determined by the Toronto consensus criteria of “probable neuropathy.” The term “probable neuropathy” implies the presence of a combination of symptoms and signs of neuropathy, which include any two or more of the following: neuropathic symptoms, decreased distal sensation, and unequivocally decreased or absent ankle reflexes [29]. The definition of neuropathy above by the Toronto consensus criteria was with regard to typical DPN, and atypical DPN includes painful, autonomic, and nerve morphologic abnormalities (small fibre neuropathy). In typical DPN, autonomic dysfunction and neuropathic pain may also develop over time. Therefore, these symptoms were checked in all patients (see Section 2.3). In this paper, the term “DPN” principally signifies “diabetic symmetric sensorimotor polyneuropathy” but implies also part of diabetic autonomic neuropathy.

Diagnosis of diabetic retinopathy was made by an indirect ophthalmic examination based on the presence of clinical features in the fundus of both eyes. Diabetic retinopathy was subdivided as follows: no apparent diabetic retinopathy, simple diabetic retinopathy, preproliferative diabetic retinopathy, and proliferative diabetic retinopathy [30]. Those who had received panretinal photocoagulation were included in the category of proliferative retinopathy. Retinopathy was defined when simple diabetic retinopathy was present.

Diabetic nephropathy was classified into four groups (patients receiving dialysis therapy were excluded) as follows: (1) prenephropathy with normoalbuminuria (estimated glomerular filtration rate (eGFR) ≥ 30 mL/min/1.73 m^2^), (2) incipient nephropathy with microalbuminuria (eGFR ≥ 30 mL/min/1.73 m^2^), (3) overt nephropathy with macroalbuminuria or persistent proteinuria (eGFR ≥ 30 mL/min/1.73 m^2^), and (4) kidney failure with any albuminuria/proteinuria status (eGFR < 30 mL/min/1.73 m^2^), according to a new classification of diabetic nephropathy by a joint committee on diabetic nephropathy in Japan in 2014 [31]. Microalbuminuria was defined as an abnormally increased excretion rate of albumin in the urine in the range of 30–299 mg/g creatinine. Nephropathy was defined when microalbuminuria was present.

### 2.2. Physical Examination and Laboratory Measurements

For every patient, general medical assessments, such as body height, body weight, blood pressure, medical history taking, and blood sampling, were performed. The body mass index was derived from the following calculation: body weight (kg) divided by the square of body height (m). Blood pressure was measured in the supine position twice in a quiet room using an automated sphygmomanometer, and the mean levels were recorded [32]. Mean blood pressure was approximated by calculating the following formula: (systolic blood pressure − diastolic blood pressure)/3 + diastolic blood pressure. Hypertension was defined as blood pressure above 140/90 mmHg or taking antihypertensive medication. In medical history taking, smoking status and the duration of diabetes were recorded. Blood samples were collected in the morning after 12 hours of fasting. Haemoglobin A1c (HbA1c) levels were measured by a high-performance liquid chromatography method. The present study presented HbA1c of the National Glycohaemoglobin Standardization Program (NGSP) equivalent value (%) together with the International Federation of Clinical Chemistry unit (mmol/mol) converted from NGSP value. Fasting plasma glucose, total cholesterol (TC), high-density lipoprotein cholesterol (HDL-C), and triglyceride (TG) levels were determined by enzymatic assays [33–36]. Low-density lipoprotein cholesterol (LDL-C) levels were calculated according to the Friedewald formula: LDL − C = TC − HDL − C − TG/5. Cystatin C levels were measured by latex agglutination turbidimetry [37].

### 2.3. Estimation of Diabetic Neuropathy

Every patient was asked about neuropathic symptoms. These symptoms included somatic symptoms, such as bilateral numbness, tingling, and a prickling sensation, and dysesthesia of the toes and soles of both of the feet. Additionally, autonomic symptoms, such as orthostatic hypotension, persistent constipation, and diarrhea, were recorded.

For neurological signs, the Achilles tendon reflex was checked. This reflex was regarded as decreased when there was a response for both legs in the knee-standing position (reinforcement method) with a percussion hammer or as disappeared when there was no response, even with such a reinforcement method. Vibratory perception was tested using a 128 Hz tuning fork. Shortening of the perceptible time for a fork vibration of less than 10 seconds was judged as attenuation of vibration perception. Semmes-Weinstein monofilament tests were performed to identify the presence of anesthesia. The patients lay down and closed their eyes. Monofilaments were then pressed onto the top and bottom of both feet until the tips of the filaments started to bend, changing the diameter of the filaments. When filaments that were thinner than number 3.84 were imperceptible, the patients were considered as having sensory attenuation. Muscle weakness and atrophy were estimated by a manual muscle test for the extensor digitorum brevis muscle in both feet. All these neurological symptoms and signs were arranged and fitted into 5 diabetic neuropathy stages by the Diabetic Neuropathy Study Group in Japan [38]: (I) no signs and symptoms or one of the following: bilateral foot symptoms, bilateral attenuation of Achilles tendon reflex, and bilateral attenuation of vibration perception; (II) bilateral attenuation of Achilles tendon reflex and bilateral attenuation of vibration perception; (III) II plus bilateral foot symptoms and bilateral attenuation of touch sensation; (IV) III plus autonomic symptoms (orthostatic hypotension, persistent diarrhea, and persistent constipation); and (V) IV plus muscle atrophy or weakness of extensor digitorum brevis (maybe without bilateral foot symptoms). Signs (Achilles tendon reflex > vibration test and monofilament) and the results of nerve conduction studies are given priority over somatic and autonomic symptoms when determining the stage due to the insufficient reliability of patients' statement about their symptoms.

The CV_R-R_ at rest and in deep breathing was measured. After the patient had rested in the supine position for at least 10 minutes, a standard 12-lead electrocardiogram was recorded (Cardio StarFCP-7541; Nihon Kohden Corporation, Tokyo, Japan). The R-R intervals were measured for 100 heartbeats at resting breathing and then for 100 heartbeats while taking deep breaths (1 time for 5 seconds). The CV_R-R_ was obtained by dividing the standard deviation (SD) by the mean (M): CV (%) = (SD/M) × 100 [39].

For sural nerve sensory conduction velocity, an active electrode was positioned on the middle point between the lateral malleolus and the tip of the little toe. A reference electrode was placed 3 cm distal to the active electrode. A ground electrode was placed on the distal lateral leg between stimulating and recording electrodes. For stimulation, a cathode was placed between the lateral malleolus and the Achilles tendon.

For peroneal nerve motor conduction velocity, an active electrode was positioned over the belly of the extensor digitorum brevis muscle. A reference electrode was placed distal to the active electrode over the tendon of the extensor digitorum brevis muscle. For stimulation, a cathode was placed distally at the middle point between the Achilles tendon and lateral malleolus and proximally above the head of the fibula.

For tibial nerve motor conduction velocity, an active electrode was positioned over the belly of the abductor hallucis muscle slightly beneath and in front of the navicular bone. A reference electrode was placed distal to the active electrode over the tendon of the abductor hallucis. For stimulation, a cathode was placed distally on the point proximal to the active electrode between the medial malleolus and the Achilles tendon and proximally slightly lateral to the center of the skin line in the popliteal fossa.

For median nerve F-wave conduction velocity, active electrodes were positioned over the belly of the abductor pollicis brevis muscle. A reference electrode was placed over the tendon of the abductor pollicis brevis at the base of the phalanx of the thumb. For supramaximal stimulation for the F-wave, a cathode was placed between the tendon of the palmaris longus muscle and that of the radial flexor muscle of the wrist. The distance was measured connecting the wrist, elbow, axillary line, middle point of the clavicle, and seventh cervical spinous process.

Sensory, motor, and F-wave conduction velocities of the nerves, of which action potentials could not be obtained, were omitted. Omitted numbers are 24, 19, 1, and 5 for sural sensory, peroneal motor, tibial motor, and median F-wave conduction velocities, respectively.

In all NCS measurements, a ground electrode was placed on the dorsum of the hand and foot between stimulating and active (recording) electrodes. The anode was 2 cm proximal to the cathode. All of the subjects were positioned in a quiet room and remained awake while testing. Skin temperature was maintained above 33°C in the upper limbs (at the midpoint of the forearm) and above 32°C in the lower limbs (at the midpoint of the lower leg), as measured by a far infrared ray thermometer.

The measuring device that was used for the NCS was the NeuropackM1 KD-026A® (Nihon Kohden Corporation).

### 2.4. Measurement of the CAVI

The subjects were placed in the supine position. Blood pressure was measured at the brachial artery, and heart sounds were monitored using an electrocardiogram. The length from the aortic valve to the ankle and the time taken for the pulse wave to propagate from the heart to the ankle were measured. The pulse wave velocity from the heart to the ankle was obtained by dividing the length from the aortic valve to the ankle by the time taken for the pulse wave to propagate from the heart.

The principle of the CAVI formula and its calculation have been described previously [23]. For statistical evaluation of the CAVI, mean values of the left and right sides were used. The CAVI was measured by the VaSera VS-1000 (Fukuda Denshi, Tokyo, Japan).

### 2.5. Statistical Analysis

Statistical analysis was performed using Stata SE® ver. 12 (StataCorp LP, College Station, TX, USA). Clinical profiles of the recruited patients are shown as mean ± standard deviation. Simple and multiple linear regression analyses were performed to investigate determinants of neuropathy indices and the CAVI. Biologically plausible predictors were as follows: age, sex, presence of obesity, mean arterial pressure, presence of dyslipidaemia (defined as TG levels > 3.9 mmol/L, HDL-C levels < 1.0 mmol/L, LDL-C levels > 3.6 mmol/L, or taking medications for dyslipidaemia), current smoking, cystatin C, and HbA1c. Obesity was defined as body mass index (BMI) ≥ 25 kg/m^2^ [40]. Cystatin C is a kidney function marker independent of sex, age, and muscle mass compared with serum creatinine [41]. Multilinearity was checked by the variance inflation factor.

## 3. Results

### 3.1. Clinical Profiles of the Patients

Most patients were middle-aged to older people, and 64.5% were male. The mean duration of diabetes was 13.0 ± 8.2 years. The rates of hypertension and dyslipidaemia were 48.2% and 73.5%, respectively. Those who had a habit of drinking, excluding chance drinkers, comprised 35.5% of the patients, but there were no alcohol addicts, leading to alcoholic neuropathy. Microangiopathies, such as retinopathy and nephropathy, were detected in approximately 40–50% of all patients (Table 1).

The CAVI value less than 8.0 was estimated as normal. The mean CAVI value of this population was 8.6 ± 1.4 and only 31.3% was within normal range. Somatosensory symptoms of both feet were observed in only 31.9% of patients, but the Achilles tendon reflex, vibration perception, and tactile sense of pressure as assessed by monofilaments were attenuated or lost in 54.5%, 41.6%, and 38.8% of patients, respectively. The effect of aging cannot be excluded when assessing these signs. Autonomic symptoms were detected in 56.6% of all patients, although these symptoms cannot clearly be attributable to DPN. Only 13.3% of the patients had atrophy and muscle weakness of the extensor digitorum brevis muscle. Neuropathy was diagnosed in 57.6% of all patients by the Toronto consensus criteria. There was a wide variation in the neuropathy stages in this population, thus considered suitable for the statistical analyses (Table 2).

### 3.2. Simple and Multiple Linear Regression Analyses between Indices of Diabetic Neuropathy and Physiological Parameters for Arteriosclerosis

Simple linear regression showed that the CAVI was significantly associated with sural sensory nerve conduction velocity and median F-wave conduction velocity (*p* < 0.001; *p* < 0.001).

Multiple linear regression of other neuropathies and risk factors of arteriosclerosis (age, sex, presence of obesity, body height, mean arterial pressure, presence of dyslipidaemia, current smoking, cystatin C, and HbA1c) showed that the CAVI was significantly associated with sural sensory nerve conduction velocity and median F-wave conduction velocity (Table 3).

In multiple linear regression analysis for the CAVI, sural nerve conduction velocity remained a significant variable after the variable of age (*p* < 0.001). The same analysis for sural nerve conduction velocity showed that the CAVI remained the most significant explanatory variable (*p* < 0.001). The same analysis for the CAVI showed that median nerve F-wave conduction velocity remained a significant explanatory variable following age (*p* = 0.001). The same analysis for median nerve F-wave conduction velocity showed that the CAVI remained a significant explanatory variable following HbA1c (*p* = 0.001).

There was no multilinearity between explanatory variables (Table 4).

## 4. Discussion

In the current study, multiple linear regression analysis for sural sensory nerve conduction velocity and median nerve F-wave conduction velocity showed that the CAVI remained a significant explanatory variable in its relation to diabetic neuropathy and vice versa. Previous studies have shown that CAVI values differ between those with neuropathy and those without neuropathy [24, 25]. The present study evaluated and identified the relation of the CAVI with neuropathy by direct statistical assessment with objective measurement of DPN (using the NCS).

NCS markers of the lower extremities are primarily used for assessment of diabetic neuropathy. The most representative abnormalities of nerve conduction in DPN are peroneal motor nerve conduction velocity, compound muscle action potentials, distal latency, sural sensory nerve conduction velocity, sensory action potentials, and tibial distal latency [26]. Our study was conducted before normalization of nerve conduction measurements, such as the location of electrodes, in our institute. Therefore, distal latency and amplitude were not adopted. F-wave studies can be used to assess conduction of proximal nerve segments in contrast to conduction of only the distal segments in routine NCSs. Therefore, we measured conduction velocity of the median nerve. F-wave conduction velocity of the tibial nerve was not measured because of the potential uncertainty of determination of distance. The F-wave is due to direct antidromic activation of spinal motor neurons. The same motor axon serves as the afferent and efferent arc. Sural sensory nerve conduction velocity, which was a determinant of the CAVI in our study, was selected among NCS parameters by a newly developed automated NCS device (NC-stat DPNCheck®; Waltham, Massachusetts, USA) for screening, early detection, and diagnosis of DPN together with sural nerve action potentials [42]. The reason why peroneal motor nerve conduction velocity had no relation with the CAVI may be partly due to the potential compression damage caused by the habit of sitting squarely in the elderly Japanese population, but the exact reason for this poor regression remains unknown.

A decrease in vascular elasticity (i.e., arterial stiffness) mainly depends on an abnormal increase and thickening of the intima. Subsequently, elastic fibres of the media in the elastic artery (not in muscular-type arteries) rupture, which leads to a loss of extensibility of the vascular wall. Elastic arteries play an auxiliary role in the pumping function of the heart. These arteries also help to alter intermittent cardiac ejection of continuous blood flow to muscular arteries and small vessels. Loss of vascular elasticity is due to a decrease in elastin and an increase in collagen, mainly caused by aging. This fact is supported by our finding that aging was the most significant explanatory variable in multiple regression for CAVI. Loss of vascular elasticity causes impairment of regulated continuous blood supply to the periphery and leads to ischemia and reperfusion-induced injury to neural tissues [15, 43]. This injury causes failure of autonomic drive and leads to further arteriosclerotic changes [15, 44].

In multiple regression analysis for CAVI, sural nerve conduction velocity and median nerve F-wave conduction velocity were the significant explanatory variables second to age. In the same analysis for sural nerve conduction velocity and median nerve F-wave conduction velocity, the CAVI remained the most significant explanatory variable for sural nerve conduction velocity and the second significant explanatory variable for median nerve F-wave conduction velocity following HbA1c. A study on patients with coronary artery disease showed that the CAVI could be used for predicting future cardiovascular events, although numbers of the participants were small and the included participants were limited to a specific disease [45]. These previous results suggest that the CAVI and the NCS may be related to future cardiovascular events [46].

This study has some limitations. In severe DPN cases, action potentials could not be obtained due to extinction of conducting nerve fibres. These cases are not included in this study, and analyses on NCSs, especially sural nerve sensory conduction velocity and peroneal nerve motor conduction velocity, were restricted to the cases whose conducting nerve fibres were still present. Another limitation is that this was a cross-sectional and observational study with a limited number of subjects. Therefore, a causal relationship between DPN and arteriosclerosis cannot be assumed. An interventional and large sample study is needed to further determine the pathophysiological relations between these variables. Lastly, NCSs can only assess large fibre neuropathy, and small fibre neuropathy was not fully examined. CV_R-R_ can estimate diabetic small fibre impairment, but it only measures cardiac parasympathetic nerve function and cannot be applied to patients with arrhythmia.

## 5. Conclusions

In conclusion, multiple regression analysis indicated that the CAVI, a potential predictor of cardiovascular events, remains the most significant explanatory variable for sural sensory nerve conduction velocity, lower limb NCS markers that are essential for assessment of DPN. The CAVI, an arterial stiffness and vascular damage marker, has a close relationship with sural nerve conduction velocity and median F-wave conduction velocity, a marker of distal and proximal neuropathic impairment, implying that arterial stiffness and impairment of nerve function start at a similar point of time and progress at a similar rate in patients with diabetes. When considering management of neuropathy or arteriosclerosis, assessment of one of these two complications needs to be combined with understanding of the state of the other. The NCS is an established and objective measure of DPN, but it cannot assess small fibre neuropathy, including autonomic neuropathy. Repeated failure of past clinical trials of DPN was partly attributable to this limitation [47]. Future studies concerning the relations between direct functional vascular markers and noninvasive and reliable neuropathy markers covering small fibre neuropathy are expected for early diagnosis and for preventing the advancement in severity of DPN, amputation, and cardiovascular events.

## Figures and Tables

**Table 1:** Clinical profile of the patients (*n* = 166).

Age (years)	59.1 ± 11.2
Male (%)	64.5
Body mass index (kg/m^2^)	26.6 ± 5.0
Obesity (%)	56.6
Systolic blood pressure (mmHg)	136.0 ± 17.3
Diastolic blood pressure (mmHg)	81.3 ± 14.2
Mean arterial pressure (mmHg)	99.5 ± 13.7
Hypertension (%) (above140/90 mmHg or medication)	48.2
Smoking (%)	59.0
(Ex-smoker/current smoker)	37.9/21.1
Alcohol (%) (excluding chance drinker)	35.5
Duration of diabetes (years)	13.0 ± 8.2
Retinopathy (%)	47.2 (NDR, 52.8; SDR, 22.7; PPDR, 9.2; PDR, 15.3)
Nephropathy (%)	40.4 (stage 1, 59.6; stage 2, 27.1; stage 3, 10.8; stage 4, 2.4)
Cystatin C (mmol/L)	0.72 ± 0.25
Fasting plasma glucose (mmol/L)	8.1 ± 2.5
HbA1c (mmol/mol)	67.1 ± 1.3
TG (mmol/L)	1.4 ± 0.8
HDL-C (mmol/L)	1.4 ± 0.4
LDL-C (mmol/L)(Friedewald)	2.5 ± 0.8
Dyslipidaemia (%) (TG > 3.9, HDL < 1.0, LDL > 3.6, or medication)	73.5

Values are mean ± standard deviation.

BMI: body mass index; NDR: no diabetic retinopathy; PDR: proliferative diabetic retinopathy; PPDR: preproliferative diabetic retinopathy; SD: standard deviation; SDR: simple diabetic retinopathy; TG: triglycerides; HDL-C: high-density lipoprotein cholesterol; LDL-C: low-density lipoprotein cholesterol.

**Table 2:** Clinical profile of the CAVI and DPN markers (*n* = 166).

CAVI	8.6 ± 1.4
Nerve conduction study	
Sural SCV (m/s)	42.3 ± 5.1
Peroneal MCV (m/s)	42.7 ± 4.6
Tibial MCV (m/s)	40.9 ± 4.5
Median FWCV (m/s)	61.9 ± 6.1
Neuropathy (rate of abnormality)	
Bilateral foot symptoms (%)	31.9
Decreased Achilles tendon reflex (%)	54.5
Decreased vibration perception (%)	41.6
Decreased touch sensation examined by Semmes–Weinstein monofilaments (%)	38.8
Autonomic symptoms (%)	56.6
Atrophy and muscle weakness of extensor digitorum brevis (%)	13.3
Neuropathy (by the Toronto consensus criteria) (%)	57.6
Neuropathy stage (by the Diabetic Neuropathy Study Group in Japan) (%)	1: 30.7; 2: 20.5; 3: 15.7; 4: 22.3; 5: 10.8
CV_R-R_	
Resting (%)	2.4 ± 1.3
Deep breathing (%)	4.9 ± 2.5

Values are mean ± standard deviation.

CAVI: cardio-ankle vascular index; SCV: sensory nerve conduction velocity; MCV: motor nerve conduction velocity; FWCV: F-wave conduction velocity; CV_R-R_: coefficient of variation of the R-R interval.

**Table 3:** Simple and multiple linear regression analyses of indices of diabetic neuropathy with the CAVI.

	CAVI		
	*r*	*β*	*β*'
Sural SCV	−0.41^∗∗^	−026^∗∗^	−0.36^∗∗^
Peroneal MCV	−0.16	−0.08	−0.10
Tibial MCV	−0.13	−0.13	−0.14
Median FWCV	−0.28^∗∗^	−0.25^∗∗^	−0.28^∗∗^
CVRR resting	−0.15	−0.00	−0.00
CVRR deep breathing	−0.07	0.05	0.07

Coefficients (standardized) of simple regression (*r*) and those of multiple regression (*β*) for the CAVI and (*β*') for neuropathy indices are shown. Please see the main text for details of the other explanatory variables. ^∗∗^*p* < 0.01; SCV: sensory nerve conduction velocity; MCV: motor nerve conduction velocity; FWCV: F-wave conduction velocity; CV_R-R_: coefficient of variation of the R-R interval.

**Table 4:** Standardized coefficients of multiple linear regression (*β*, *β*') of all of the explanatory variables.

CAVI	*β*
Sural SCV/median FWCV	−0.26^∗∗^/−0.25^∗∗^
Age	0.41^∗∗^/0.37^∗∗^
Gender	−0.02/0.02
Obesity	−0.21^∗∗^/−0.13
Mean arterial pressure	−0.02/−0.01
Dyslipidaemia	−0.06/−0.10
Current smoking	0.02/0.01
HbA1c	−0.19^∗∗^/−0.18^∗^
Cystatin C	0.03/0.15
Sural SCV/median FWCV	*β*'
CAVI	−0.36^∗∗^/−0.28^∗∗^
Age	−0.05/−0.10
Gender	−0.18/−0.03
Obesity	0.16/0.21
Mean arterial pressure	−0.03/0.07
Dyslipidaemia	0.01/−0.10
Current smoking	0.12/0.01
HbA1c	−0.21^∗^/−0.34^∗∗^
Cystatin C	−0.15/−0.11

*β*: independent variables were the CAVI, ^∗^*p* < 0.05, ^∗∗^*p* < 0.01; *β*': independent variables were sural SCV and median FWCV, ^∗^*p* < 0.05, ^∗∗^*p* < 0.01; SCV: sensory nerve conduction velocity; FWCV: F-wave conduction velocity; CAVI: cardio-ankle vascular index.
